# Validation of a handheld smartphone markerless gait‐analysis tool using an estimated groundline in horses

**DOI:** 10.1002/evj.70149

**Published:** 2026-01-16

**Authors:** Karsten Key, Jakob Kirkegaard, Katja Berg, Kristian Ringkjær Andresen, Sabrina Skov Hansen

**Affiliations:** ^1^ Keydiagnostics (RealHorse®) Fredensborg Denmark; ^2^ iKeyVet® Fredensborg Denmark; ^3^ Department of Veterinary Clinical Sciences University of Copenhagen Copenhagen Denmark

**Keywords:** computer vision algorithm, equine lameness, gait analysis, Groundline, horse, Maxdiff, Mindiff, vision‐based algorithm

## Abstract

**Background:**

A handheld smartphone‐based computer vision algorithm (RealHorse® [RH]) offers accessible alternatives for equine gait analysis but requires validation against a gold‐standard three‐dimensional multicamera optical motion capture system (Qualisys® [QS]).

**Objectives:**

To evaluate the accuracy and precision of RH in measuring vertical displacement signals (VDS) at the eye, withers, back and croup in horses trotting on a straight line and on a circle.

**Study Design:**

Cross‐sectional comparative validation study of a markerless computer vision algorithm.

**Methods:**

Fifty‐nine horses were recorded while trotting on a straight line and 24 were lunged on a circle. RH detected two‐dimensional anatomical keypoints on each frame, which were used to estimate a dynamic groundline and compute ground relative VDS with stride‐based difference in maxima (Maxdiff) and minima (Mindiff). QS provided synchronous ground‐relative VDS reference values. Agreement was evaluated using mean signed error, mean absolute error and Bland–Altman analysis.

**Results:**

On the straight line (*n* = 2620 strides), the pooled stride‐level MAE for Maxdiff and Mindiff was 3.8 mm. Keypoint‐specific errors were 5.1 mm (eye), 4.3 mm (withers) and 3.0 mm (croup). On the circle (*n* = 2419 strides), pooled stride‐level error increased to 5.5 mm. Trial‐level analysis (*n* = 58 trials) showed much lower errors: 1.4 mm for both eye and withers and 1.1 mm for croup. On the circle (*n* = 24 trials), trial‐level errors were higher, with 2.8 mm for the eye, 1.8 mm for the withers and 3.3 mm for the croup. The back keypoint consistently showed the lowest errors across both stride and trial levels.

**Main Limitations:**

RH measurements of the croup Mindiff during circling resulted in higher values and showed the largest error.

**Conclusions:**

RH measured vertical displacement of all keypoints with high accuracy and precision (trial‐level MAE 1.1–1.4 mm straight, 1.8–3.3 mm circle), supporting its use for equine gait analysis.

## 1 INTRODUCTION

Equine lameness remains a frequent diagnostic challenge in clinical practice.^1^ Accurate gait evaluation is central to effective case management. Expert visual examination is valuable, but it is also vulnerable to bias and to inter‐ and intra‐observer variability^2^, ^3^, ^4^, ^5^, ^6^ and a cause of professional insecurity for many veterinarians.^7^ Objective technologies such as force plates, inertial sensors and optical motion capture can provide precise measurements.^8^, ^9^, ^10^, ^11^ These systems often require specialised and expensive equipment as well as controlled environments, which limits their day‐to‐day use in the field. Furthermore, they do not facilitate independent longitudinal use by owners, who have limited ability in detecting lameness subjectively.^12^ A recent survey among equine veterinarians specialised in lameness reported that early detection of asymmetry could prevent lameness^7^ and horse owner performed longitudinal gait analysis is therefore increasingly relevant.

Interest in objective gait analysis and biomechanics has increased in recent years.^13^ A well‐established approach is to track the cyclical vertical motion for key anatomical points during trot, most notably the head, withers and pelvis, because this captures overall symmetry and helps detect lameness.^8^, ^9^, ^14^, ^15^ Within each stride, indicators of impact and push off asymmetry, Mindiff and Maxdiff, are quantified by the difference in the vertical displacement signal (VDS) between the two minima and maxima, respectively. These symmetry metrics are implemented across platforms including inertial systems, optical motion capture and computer vision tools, which underlines their central role in data driven gait assessment.^8^, ^9^, ^14^, ^16^, ^17^, ^18^ Advances in computer vision and deep learning now allow markerless two‐dimensional motion capture from ordinary video.^19^, ^20^, ^21^, ^22^, ^23^, ^24^, ^25^, ^26^ By locating anatomical landmarks such as head, withers and croup as pixel coordinates frame by frame, these systems derive the two‐dimensional cyclical VDS and thereby offer a more accessible option for clinicians and for owners.^19^, ^26^ The robustness of the algorithm used in this study has been investigated under the varied and imperfect conditions of field work,^27^ and the effect of handheld recording and the effect of using a dynamic estimated groundline with the same system have been investigated in a treadmill study.^19^

Objective systems generally follow one of two strategies. One approach derives a VDS referenced to the ground in metric units,^19^, ^27^ which is the ground relative method used in this study. A second approach estimates asymmetry as a ratio by relating the first and the second harmonics of the signal.^8^, ^9^, ^14^, ^15^, ^16^, ^17^, ^18^, ^26^, ^28^, ^29^ The aim of the study was to validate a custom two‐dimensional (2D) markerless computer vision algorithm (RH) against a multi camera three‐dimensional (3D) optical motion capture system (QS) for equine gait analysis in horses trotting on a straight line and on a circle.

## 2 MATERIALS AND METHODS

**FIGURE 1:**
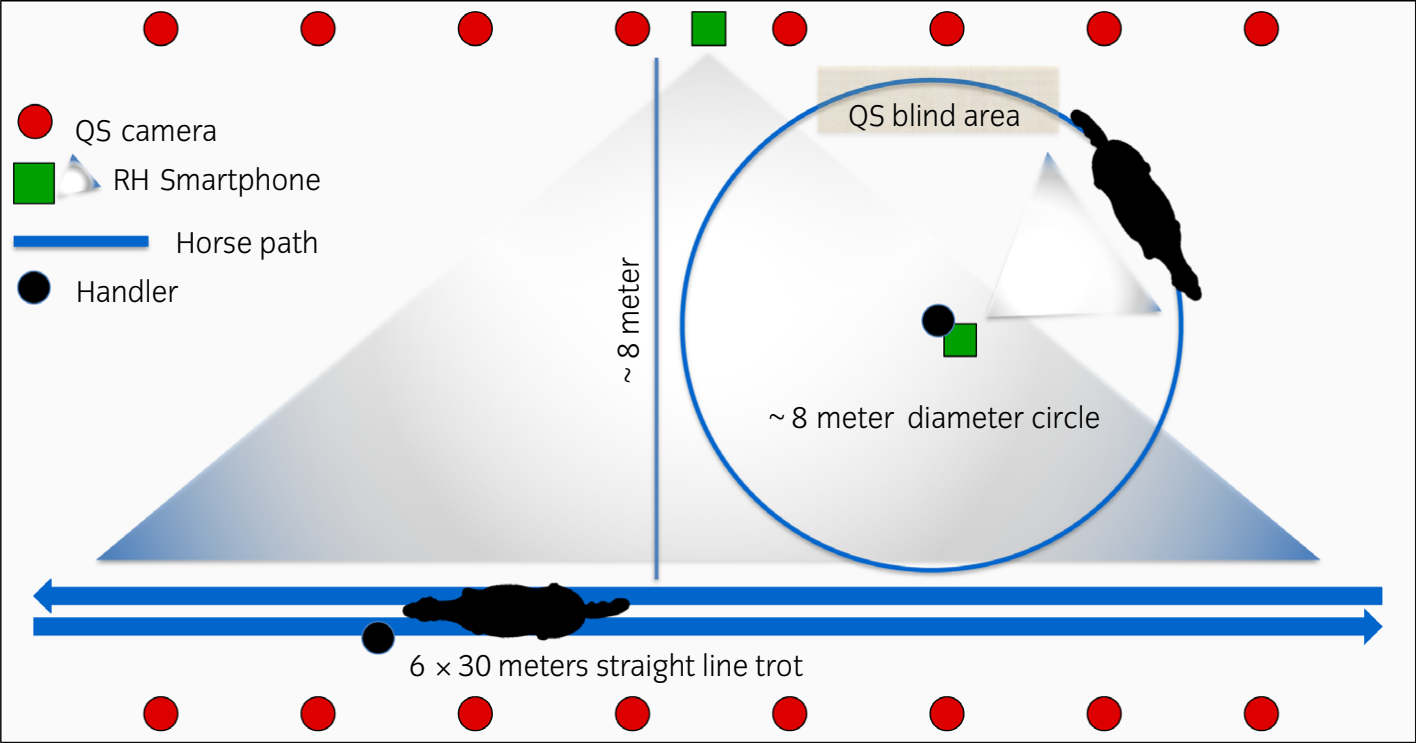
Experimental setup. Horses were recorded while trotting on a straight line and on a circle. On the straight line the smartphone camera was handheld 8 m from the line of trot. While lungeing, the distance from the handheld camera to the horse was approximately 4 m.

We conducted a comparative study to benchmark a 2D markerless computer vision algorithm (RH) (RealHorse®) against a 3D optical motion capture reference (QS) (Qualisys®) (72/100 Hz) (Figure 1). Side view smartphone videos were recorded handheld with the standard settings, high definition (HD) and 30 frames per second (FPS) (iPhone 15 Pro Max [iOS version 18.6.2, Apple Inc]) (30 Hz). A backup Camera B was also used for the straight‐line recordings, but none of the data from this camera was subsequently used for comparative analysis. The 2D and 3D streams were synchronised with a light cue visible to both systems, providing frame‐level precise alignment. For both systems, we produced VDSs in metric units for the eye, withers and croup and evaluated agreement at stride and trial level using the symmetry metrics Maxdiff and Mindiff.

### 2.1 Study population and test conditions

Fifty‐nine horses were enrolled in the study (26 warmbloods, 11 standardbred trotters, 6 Icelandic horses, 3 Irish cross, 3 ponies, 2 Thoroughbreds, 2 Shetland Ponies, 1 Fjordhorse cross, 1 Irish cob, 1 PRE, 1 pinto, 1 pinto pony, 1 warmblood cross yearling). Mean age was 11.4 years (range 1–28 years), mean height at the withers 157 cm (range 92–179 cm) and mean bodyweight 507 kg (range 170–686 kg). Coat colours included bay, black, red, grey, yellow and pinto. One Icelandic horse was excluded from the study because it would not trot. None of the horses included in this study contributed training data to the RH algorithm.

The horses were trotted six times over a 30‐m straight line on rough, level concrete, without prior practice runs. They represented a convenience sample of client‐owned horses from one clinic and from the university teaching herd, which were brought for inclusion if considered sound or only mildly lame by their owners or university personnel. According to the protocol, an experienced equine lameness specialist, certified by both the International Society of Equine Locomotor Pathology and the Equine Gait Analysis Society, was instructed to exclude any horse subjectively graded as 3 or higher on a 0–5 scale, where 0 represents soundness and 5 denotes non weightbearing lameness. Some horses did display asymmetries within the range of mild lameness, but all were able to complete straight‐line trials. Lungeing on the rough concrete was performed in a subset of 24 of 59 horses, only when the owners provided explicit verbal consent for this and when horses were fit and expected to be calm on the lunge. Horses that did not trot comfortably and calmly on the circle were stopped immediately. Consequently, the duration and stride distribution between left and right circles varied.

A hard and level surface was required for both straight line and circle measurements because we were validating the RH system, which estimates vertical displacement relative to a dynamically estimated groundline,^19^ against the QS gold‐standard method that measured vertical displacement relative to the calibrated ground plane. Even small irregularities on the surface would therefore introduce error into the reference signal. Agreement of the RH system under less controlled field conditions on soft riding surfaces has been investigated in a separate study.^27^

### 2.2 Motion capture (QS) marker detection

The 3D multi‐camera optical motion capture system (QS) served as the reference and used 16 cameras and recorded at either 72 or 100 hertz. The choice between which frequency varied across sessions due to a setting error and was not assigned according to any study factor. Calibration of the capture volume was performed by QS personnel according to the manufacturer procedure. The system recorded the 3D trajectories of reflective markers at the forehead (between eyes), withers, back (lowest position) and croup (between both tuber sacrale) locations. After universal calibration of all QS data to align the *x*‐axis and *y*‐axis to the level concrete floor, the *z*‐axis VDSs were extracted.

### 2.3 Algorithm RH‐detected keypoints

Two dimensional videos were acquired in landscape orientation and HD at 30 frames per second using a handheld iPhone and the standard camera settings. On the straight line the camera was handheld approximately 8 m from the line of trot. While lungeing, the approximate camera to horse distance was 4 m, handheld by a person standing next to the person lungeing the horse (Figure 1).

**FIGURE 2:**
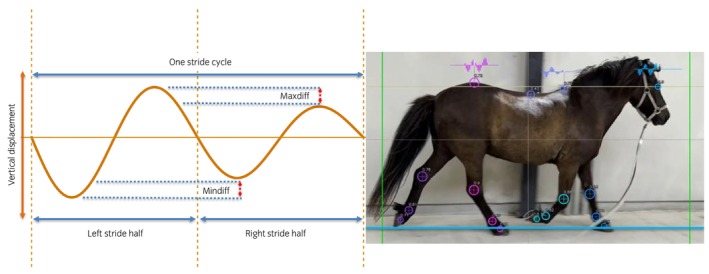
Left: Filtered vertical displacement signals from the eye, withers, back, and croup during one stride of trot. Mindiff and Maxdiff are computed per stride as the differences between the two minima and two maxima, respectively. In this example on the left, both Mindiff and Maxdiff parameters indicate a deficit on the right limb. Right: Detected keypoints (circles). Graphs over the croup, withers and eye (pink, purple, blue) show relative motion to the dynamically estimated groundline (light blue).

Keypoint detection in the 2D videos was performed by a custom made and trained deep neural network (RH) designed to identify anatomical landmarks from a side view. The model includes multiple convolutional layers that extract spatial features, followed by sequential refinement of keypoint locations to enhance accuracy. The primary keypoints were the eye, withers, back and croup, along with limb and hoof positions used to estimate the dynamic groundline (Figure 2). The network processes each frame and estimates keypoint positions with sub‐pixel precision, generating time‐series displacement signals used for subsequent stride‐ and trial‐based analysis.

### 2.4 Anatomical RH and QS keypoint variations

The keypoints were equivalent at rest for withers, back (at lowest back position) and croup (between both tuber sacrale). The lowest point of the back shifts horizontally during trot. QS detects the marker placed at rest, whereas RH identifies the dynamically lowest point.

For the croup viewed by RH, the highest point over the croup position can however sometimes be the gluteus muscle rather than the croup (both tuber sacrale) itself, and the RH algorithm will select the highest point at the croup position. For the RH eye keypoint, QS used a single marker placed on the forehead between the eyes, while the RH algorithm always detects the eye that faces the camera.

### 2.5 Stride exclusion

The RH algorithm is designed to extract strides only when the median withers‐to‐croup distance exceeds 40% of the trial 95th percentile. This criterion excludes strides where the horse is viewed at an oblique angle of approximately <40° or >140°. In this study, the straight‐line trotting area covered by the QS system was within this viewing range and approximately 30 m long. Additional algorithm built‐in data sanity checks that trigger stride exclusion are VDSs exceeding 30% of withers height, minima higher than maxima (and vice versa) or stride durations deviating >30% from the trial median. Lastly, if an analysed keypoint was not present in each frame, stride detection for that frame and keypoint would fail. These built‐in exclusion criteria lead to minor differences in the number of detected strides across keypoints. No RH data curation was performed outside the algorithm, and hence no strides were excluded based on within‐trial RH variation.

Strides with incomplete motion capture (QS) graphs were identified automatically and removed before comparative analysis. The lungeing area had incomplete QS coverage at one periphery (Figure 1), and because the system requires an unobstructed view of each marker by at least three cameras, some QS stride detections were missing or incomplete.

### 2.6 Signal filtering and symmetry metrics computation

For the RH algorithm the dynamic groundline was estimated frame by frame from the hoof keypoints by identifying stance and swing intervals^8^, ^9^, ^14^, ^30^ and applying proprietary RH algorithm processing. Two‐dimensional videos were stabilised using the estimated groundline and frames were rectified before further analysis. Recordings were calibrated to millimetres using the known withers height so that vertical motion could be expressed in metric units. In the rectified videos the eye, withers, back and croup were evaluated relative to the estimated groundline to generate the metric VDS (Figure 2). The QS markers VDSs, referenced to the calibrated global z‐axis perpendicular to the level concrete floor, were extracted directly from the software (Qualisys®, QTM).

The raw signal from both systems combined trotting content with low frequency drift from 2D camera motion (RH), keypoint or marker detection fluctuation (RH/QS) or marker skin movement (QS) and occasional erratic horse movement. To isolate the relevant frequency band, the expected trotting frequency was estimated from stride timing from the RH algorithm, and a third‐order Butterworth high‐pass filter was applied with a cut off set just below the trial estimated trot frequency.^15^, ^31^ Once the filtered signal was obtained, strides were segmented using expected peaks and valleys with guidance from hoof timing for stance and swing from the RH signal.^29^ The RH algorithm had further proprietary postprocessing steps, but without exclusion of strides based on VDS variation. Symmetry was quantified by Maxdiff and Mindiff for each stride following the definitions of Keegan et al.^16^ These metrics were compared between the RH algorithm output and the QS reference for the eye/forehead, withers, back and croup on the straight line and, where feasible, on the circle.

### 2.7 Data analysis

We performed a stride‐ and trial‐based comparative analysis using the Maxdiff and Mindiff variables extracted from the two systems. We summarised agreement between the RH output and the QS reference using descriptive statistics. For each comparison we calculated the mean absolute error (MAE) as a measure of precision, the mean signed error (MSE) as a measure of bias and the sample standard deviation. We also reported limits of agreement for the signed error and confidence intervals for the MAE.

Bland Altman plots and scatterplots were produced to visually evaluate agreement between the RH algorithm and the QS reference at stride level and at trial level.^32^, ^33^ Bland Altman analyses were performed at two levels. At the trial level, each observation represented independent trials per horse and condition, and limits of agreement were calculated and interpreted in the usual way. At the stride level, our aim was to assess the algorithm's accuracy for individual strides. Because stride‐level Maxdiff and Mindiff reflect a changing signal with high within‐trial variation from stride to stride, each stride was treated as a separate observation, corresponding to the non‐constant repeated‐measure situation described by Bland and Altman.^34^ Visual inspection of the plots did not indicate clustering of the data points around trials. Limits of agreement were therefore calculated in the standard manner, but the stride‐level Bland Altman plots are interpreted descriptively, as correlations within horses may slightly reduce data independence, meaning the true limits of agreement (LoA) interval could be slightly wider.^34^

Distributions of signed and absolute errors were examined with histograms to describe clustering, skewness and tail behaviour. All analyses were performed separately for the eye/forehead, withers, back and croup, and were stratified by movement either on a straight line or on a circle.

## 3 RESULTS

### 3.1 Stride detection and missing data

Across straight line and circle trials, a total of 5039 strides were detected by the RH algorithm: straight line, 2620; left circle, 1658; right circle, 761.

All videos were reviewed for missing RH keypoint/stride detections at the eye, withers and croup. On the straight line, 132 strides missed detection at one of the three keypoints: eye, 34; withers, 40; croup, 58. On the circle, 11 keypoint/strides failed detection: eye, 1; withers, 1; croup, 9. Overall average RH keypoint/stride detection failure was 1.7% on a straight line and 0.2% on a circle.

RH‐detected strides with incomplete QS motion capture graphs were removed. The QS reference failed to produce complete keypoint stride data in 696 instances: eye/forehead, 389; withers, 119; back, 100; croup, 88.

### 3.2 Stride‐based results

**FIGURE 3:**
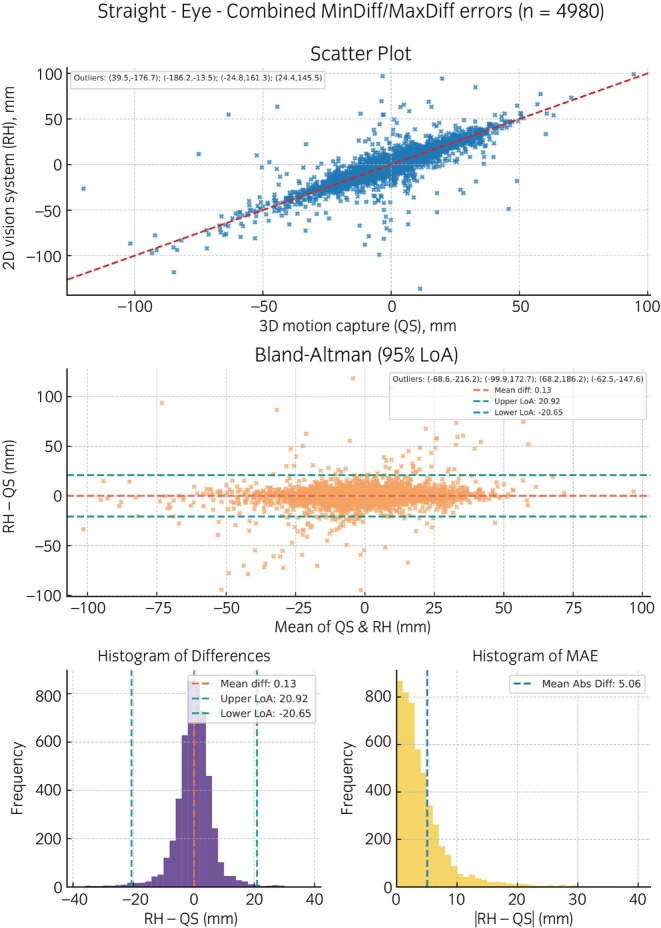
Stride‐level Maxdiff and Mindiff aggregated data comparing RH and QS for the Eye on a straight line. The scatter plot in the top panel shows the paired values against the line of equality. The Bland Altman plot in the middle panel shows the bias of 0.13 mm and the 95% limits of agreement at −20.65 to +20.92 mm. The histogram of absolute differences shows a mean absolute error (MAE) of 5.06 mm. Observations include *n* = 4980 paired stride‐level metrics. Stride‐level limits of agreement (LoA) are indicative and should be interpreted descriptively because strides within a horse are not fully independent.

**FIGURE 4:**
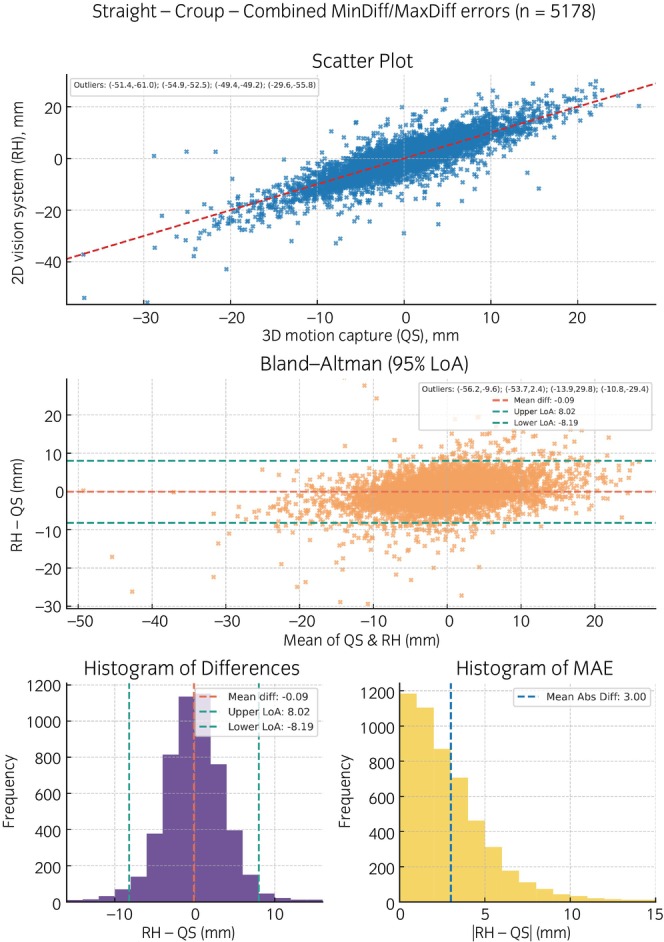
Stride‐level Maxdiff and Mindiff aggregated data comparing RH and QS for the Croup on a straight line. The scatter plot in the top panel shows the paired values against the line of equality. The Bland Altman plot in the middle panel shows a bias of −0.09 mm with 95% limits of agreement at −8.19 to +8.02 mm. The histogram of absolute differences shows a mean absolute error (MAE) of 3.00 mm. Observations include *n* = 5178 paired stride‐level metrics. Stride‐level limits of agreement (LoA) are indicative and should be interpreted descriptively because strides within a horse are not fully independent.

Agreement metrics included MAE, MSE, sample standard deviation, confidence intervals and limits of agreement. Tables 1 and 2 report the number of paired strides and corresponding errors for Maxdiff and Mindiff on the straight line and circle. Figures 3 and 4 present the straight line combined Maxdiff and Mindiff error plots for eye and croup keypoints. Additional keypoint error plots can be found in Figures [Link], [Link].

**TABLE 1:** Stride‐based analysis of vertical displacement difference (Maxdiff or Mindiff) measured at four anatomical locations (eye, withers, back and croup) during trot on a straight line.

Anatomical location (Keypoint)	Metric (vertical displacement difference)	*n* (stride‐level metrics)	MAE	95% CI	MSE	SD	95% LoA
All keypoints	Maxdiff	10,251	**3.64**	[3.52; 3.76]	−0.05	7.23	[−14.22; 14.13]
	Mindiff	10,251	**3.92**	[3.80; 4.03]	0.02	6.96	[−13.62; 13.67]
Eye	Maxdiff	2490	**4.71**	[4.35; 5.07]	0.25	10.28	[−19.89; 20.39]
	Mindiff	2490	**5.41**	[5.04; 5.78]	0.02	10.92	[−21.39; 21.42]
Withers	Maxdiff	2583	**4.26**	[3.97; 4.55]	−0.11	8.66	[−17.08; 16.85]
	Mindiff	2583	**4.36**	[4.18; 4.54]	−0.10	6.36	[−12.55; 12.36]
Back	Maxdiff	2589	**2.76**	[2.65; 2.87]	0.01	3.97	[−7.78; 7.80]
	Mindiff	2589	**2.80**	[2.68; 2.92]	0.03	4.21	[−8.22; 8.27]
Croup	Maxdiff	2589	**2.86**	[2.76; 2.96]	−0.32	3.86	[−7.89; 7.25]
	Mindiff	2589	**3.15**	[3.03; 3.27]	0.15	4.38	[−8.44; 8.73]

*Note*: The table shows mean absolute error (MAE, including 95% confidence intervals [CI]), mean signed error (MSE), standard deviation (SD) and 95% limits of agreement (LoA) for each keypoint. Stride‐level LoA should be interpreted descriptively because strides within a horse are not fully independent. Data were obtained from 58 horses (10,251 stride‐level metrics in total). Bold indicates the important metric.

**TABLE 2:** Stride‐based analysis of vertical displacement difference (Maxdiff or Mindiff) measured at four anatomical locations (eye, withers, back, and croup) during trot on circles.

Anatomical location (Keypoint)	Metric (vertical displacement difference)	*n* (stride‐level metrics)	MAE	95% CI	MSE	SD	95% LoA
All keypoints	Maxdiff	9164	**5.46**	[5.31; 5.62]	−0.63	9.25	[−18.75; 17.49]
	Mindiff	9164	**6.43**	[6.29; 6.57]	0.72	9.34	[−17.59; 19.02]
Eye	Maxdiff	2131	**6.19**	[5.83; 6.55]	−1.32	10.46	[−21.82; 19.18]
	Mindiff	2131	**6.61**	[6.29; 6.92]	0.23	9.94	[−19.24; 19.70]
Withers	Maxdiff	2337	**5.59**	[5.27; 5.90]	−0.16	9.55	[−18.87; 18.56]
	Mindiff	2337	**5.60**	[5.35; 5.85]	−0.22	8.31	[−16.51; 16.07]
Back	Maxdiff	2350	**4.78**	[4.51; 5.06]	−0.46	8.23	[−16.58; 15.66]
	Mindiff	2350	**4.82**	[4.58; 5.07]	0.97	7.70	[−14.12; 16.05]
Croup	Maxdiff	2346	**5.36**	[5.08; 5.64]	−0.65	8.69	[−17.67; 16.37]
	Mindiff	2346	**8.71**	[8.43; 8.99]	1.83	10.98	[−19.69; 23.36]

*Note*: The table shows mean absolute error (MAE, including 95% confidence intervals [CI]), mean signed error (MSE), standard deviation (SD), and 95% limits of agreement (LoA) for each keypoint. Stride‐level LoA should be interpreted descriptively because strides within a horse are not fully independent. Data were obtained from 24 horses (9164 stride‐level metrics in total). Bold indicates the important metric.

On the straight line, stride‐level MAE was 3.6 mm for Maxdiff and 3.9 mm for Mindiff when all keypoints were pooled. On the circle the overall error was 5.5 and 6.4 mm for Maxdiff and Mindiff, respectively. The variation by keypoint is listed in the tables. One outlier value of 8.7 mm for croup Mindiff on the circle was noted.

### 3.3 Trial‐based results

**FIGURE 5:**
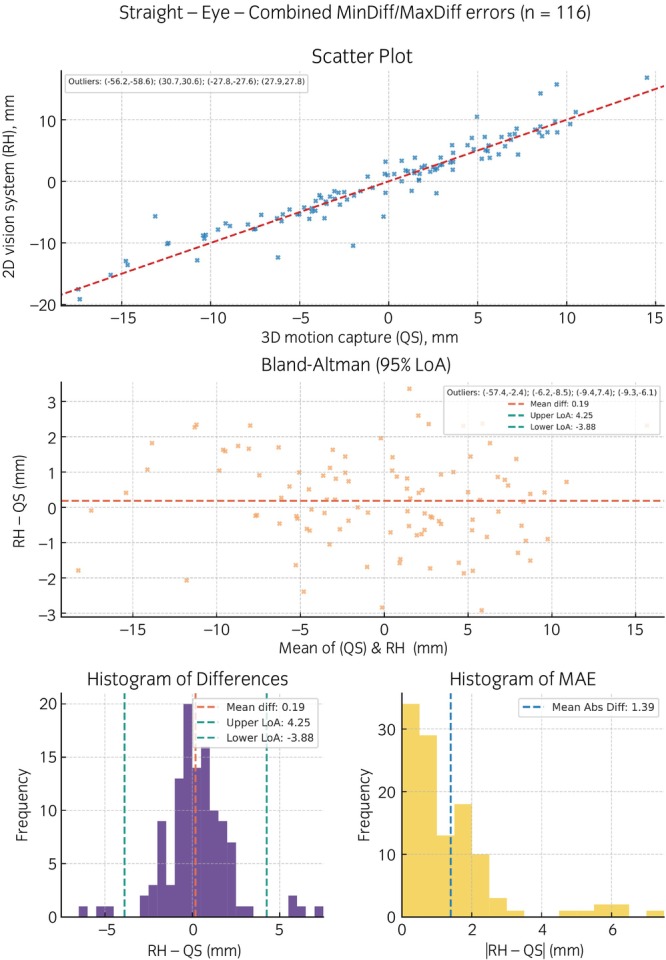
Trial‐level Maxdiff and Mindiff aggregated error for the Eye on a straight line. The scatter plot shows paired trial means against the line of equality. The Bland Altman plot shows a bias of 0.19 mm with 95% limits of agreement −3.88 to 4.25 mm. The histogram of absolute differences shows a mean absolute error (MAE) of 1.39 mm. *n* = 116 paired values.

**FIGURE 6:**
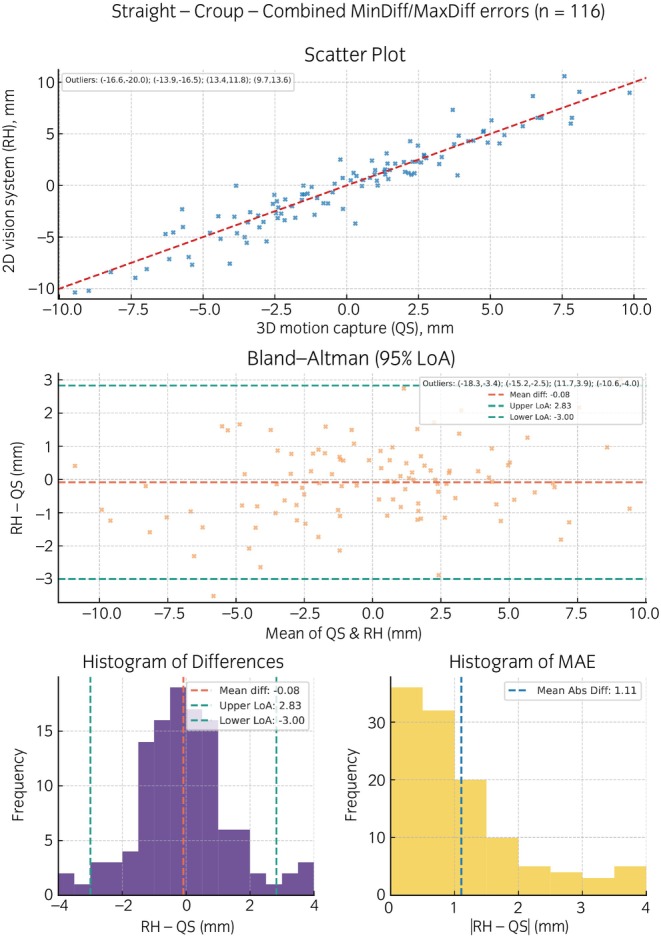
Trial‐level Maxdiff and Mindiff aggregated error for the Croup on a straight line. The scatter plot shows paired trial means against the line of equality. The Bland Altman plot shows a bias of −0.08 mm with 95% limits of agreement −3.00 to 2.83 mm. The histogram of absolute differences shows a mean absolute error (MAE) of 1.11 mm. *n* = 116 paired values.

We calculated trial‐level agreement between the RH algorithm and the QS reference for Maxdiff and Mindiff. Agreement metrics included MAE, MSE, sample standard deviation, confidence intervals and limits of agreement. Tables 3 and 4 report the number of paired trials and corresponding errors for Maxdiff and Mindiff on the straight line and circle. Figures 5 and 6 present the straight line combined Maxdiff and Mindiff error plots for eye and croup keypoints. More keypoint error plots can be found in Figures [Link], [Link].

**TABLE 3:** Trial‐based analysis of vertical displacement difference (Maxdiff or Mindiff) measured at four anatomical locations (eye, withers, back, and croup) during trot on a straight line.

Anatomical location (keypoint)	Metric (vertical displacement difference)	*n* (trial‐level metrics)	MAE	95% CI	MSE	SD	95% LoA
All keypoints	Maxdiff	232	**1.17**	[1.02; 1.33]	−0.04	1.68	[−3.34; 3.26]
	Mindiff	232	**1.17**	[1.02; 1.33]	0.01	1.69	[−3.29; 3.31]
Eye	Maxdiff	58	**1.54**	[1.13; 1.95]	0.30	2.18	[−3.96; 4.57]
	Mindiff	58	**1.25**	[0.85; 1.65]	0.07	1.98	[−3.82; 3.95]
Withers	Maxdiff	58	**1.36**	[0.98; 1.74]	−0.16	1.99	[−4.06; 3.74]
	Mindiff	58	**1.47**	[1.13; 1.81]	−0.20	1.95	[−4.02; 3.61]
Back	Maxdiff	58	**0.82**	[0.66; 0.97]	0.02	1.01	[−1.96; 2.00]
	Mindiff	58	**0.72**	[0.56; 0.89]	0.02	0.96	[−1.86; 1.91]
Croup	Maxdiff	58	**0.97**	[0.75; 1.19]	−0.33	1.24	[−2.76; 2.10]
	Mindiff	58	**1.24**	[0.95; 1.54]	0.16	1.67	[−3.11; 3.44]

*Note*: The table presents mean absolute error (MAE, including 95% confidence intervals [CI]), mean signed error (MSE), standard deviation (SD), and 95% limits of agreement (LoA) for each keypoint. Data were obtained from 58 horses (232 trial‐level metrics in total). Bold indicates the important metric.

**TABLE 4:** Trial‐based analysis of vertical displacement difference (Maxdiff or Mindiff) measured at four anatomical locations (eye, withers, back, and croup) during trot on a circle.

Anatomical location (Keypoint)	Metric (vertical displacement difference)	*n* (trial‐level metrics)	MAE	95% CI	MSE	SD	95% LoA
All keypoints	Maxdiff	96	**1.96**	[1.61; 2.32]	−0.96	2.47	[−5.80; 3.88]
	Mindiff	96	**2.65**	[2.00; 3.29]	1.02	4.01	[−6.84; 8.88]
Eye	Maxdiff	24	**2.88**	[2.10; 3.67]	−1.91	2.88	[−7.57; 3.74]
	Mindiff	24	**2.61**	[1.37; 3.86]	0.12	3.97	[−7.66; 7.91]
Withers	Maxdiff	24	**1.77**	[0.98; 2.56]	−0.38	2.57	[−5.41; 4.66]
	Mindiff	24	**1.78**	[1.13; 2.42]	−0.62	2.29	[−5.11; 3.87]
Back	Maxdiff	24	**1.39**	[0.98; 1.79]	−0.35	1.67	[−3.63; 2.94]
	Mindiff	24	**1.50**	[0.96; 2.04]	0.94	1.75	[−2.50; 4.37]
Croup	Maxdiff	24	**1.82**	[1.00; 2.64]	−1.22	2.39	[−5.89; 3.46]
	Mindiff	24	**4.70**	[2.70; 6.70]	3.63	5.62	[−7.38; 14.65]

*Note*: The table presents mean absolute error (MAE, including 95% confidence intervals [CI]), mean signed error (MSE), standard deviation (SD), and 95% limits of agreement (LoA) for each keypoint. Data were obtained from 24 horses (96 trial‐level metrics in total). Bold indicates the important metric.

On the straight line, trial‐level MAE was 1.2 mm for both Maxdiff and Mindiff when all keypoints were pooled. On the circle the overall error was 2.0 and 2.7 mm for Maxdiff and Mindiff, respectively. The variation by keypoint is listed in the tables. One outlier value of 4.7 mm for croup Mindiff on the circle was noted.

## 4 DISCUSSION

This study aimed to validate a handheld markerless computer vision system (RH) against a 3D multi camera optical motion capture (QS) reference under controlled conditions that still reflects daily clinical work. We compared symmetry metrics at stride level and at trial level for the eye, withers, back and croup on the straight and on the circle. It should be noted that vertical motion asymmetry, even when measured without error, is a diagnostic indicator rather than a definitive diagnosis.

The main finding is that accuracy and precision compared with 3D multi‐camera optical motion capture were high on the straight line at both stride and trial levels and remained well acceptable on the circle. For evaluating agreement, both stride‐ and trial‐level MAE and Bland Altman limits of agreement are relevant, as MAE describes average precision and LoA indicates the expected range of individual differences. Trial‐level agreement is the most clinically relevant, since horses are evaluated clinically over multiple strides rather than at the single stride level.

On the straight line, stride‐level MAE was 3.6 mm for Maxdiff and 3.9 mm for Mindiff when all keypoints were pooled. Trial‐level mean absolute Maxdiff or Mindiff errors were between 0.7 and 1.5 mm for each keypoint on the straight line, which is small in relation to the range of symmetry values that clinicians use for decision‐making. As expected, averaging across strides lowers trial‐level error compared with stride‐level error because random deviations cancel with repeated measurement.^27^, ^34^ The implication of this is that recording a sufficient number of strides per trial of approximately 20 strides within the RH app improves the accuracy of symmetry metrics at trial level, making the output more robust for clinical decision‐making.^27^

On the circle, stride‐level agreement was lower than on the straight, with pooled MAEs of 5.5 mm for Maxdiff and 6.4 mm for Mindiff. Trial‐level errors on the circle remained low for most keypoints (between 1.5 and 2.9 mm), although Mindiff at the croup (4.7 mm) showed a larger MAE and bias, indicating a systematic croup error at the stance phase of the stride.

The VDS amplitude of the eye (head) is approximately twice that of the other keypoints and the withers keypoint is affected by mane hair explaining higher overall stride‐level errors for these two keypoints. Back Maxdiff and Mindiff had the lowest overall error. This is promising for the use of the algorithm to detect the back's range of motion and flexion/extension patterns. This, however, needs further investigation in a focused study.

A number of limitations should be considered when interpreting these results. At the stride level, each stride was treated as a separate observation of a changing signal, which is appropriate for assessing instantaneous accuracy, but the stride‐level Bland Altman plots should be interpreted descriptively because stride metrics within a horse are not fully independent.^34^ RH stride detection failure at the eye, withers and croup was rare, occurring in only 0.2% of strides on the circle, where horses were presented in a near‐optimal side view and in 1.7% of strides on the straight line, mostly due to incorrect left versus right limb assignment by the algorithm. Back keypoint stride detection failure was not quantified as it is not shown by algorithm video output but was expected equally low.

Some random errors are present. Because the QS reference measures vertical displacement relative to the calibrated ground plane, unevenness in the surface would introduce stride‐level error in the reference signal and in comparison with the RH system. Consequently, the experiment was conducted on a hard and level surface to minimise this error, although this surface was not ideal for lungeing horses. Agreement of the system under field conditions with softer surfaces has been investigated in another study.^27^

Reflective markers used by 3D multi‐camera optical motion capture are susceptible to skin‐movement artefacts,^35^, ^36^ although such errors may be attenuated by the applied high‐pass filter.

Comparative studies of computer vision deep neural networks with systems that attach devices to the horse can be challenging, as reflective markers or other mounted sensors may introduce visual artefacts that fundamentally alter the input to the network.^37^ Visual inspection of the videos with RH markers did not reveal any apparent interaction or error between the reflective markers and the algorithm keypoint detection. To address this potential limitation, the present study was previously supplemented with a comparative analysis against manually annotated keypoints in horses trotting on a straight line and on circles under field conditions.^27^

Some systematic errors were present between the systems. First, the eye definition differs between systems. The motion capture (QS) marker was placed on the forehead between the eyes, while the vision system (RH) detects the eye that faces the camera. Head rotation in the roll axis therefore introduces projection effects in the RH signal that do not exist for the centre forehead marker, and these effects are most pronounced on the circle. Secondly, at the croup the RH algorithm selects the highest point within a predefined region. For well‐muscled horses and especially on a circle, the Gluteus muscle of the limb in stance phase can be the highest point instead of the mid sacrum, which is the location of the motion capture definition. This anatomical difference explains the larger croup Mindiff error on the circle and points to a practical way to improve future models, for example by refining the croup region of interest or by predicting a virtual sacral landmark in addition to the highest point.

The circle size was relatively small, and the surface was suboptimal for lungeing. Many of the 24 horses being lunged were more comfortable on the left rein resulting in twice as many strides on this rein, which may explain the bias for the croup Mindiff. If the data had contained an even stride distribution between left and right circle the bias would most likely have cancelled each other out and the absolute trial error would have improved. Stride absolute error would have been unaffected by this change. This is very much like the real‐world clinical situation where circle bias and systematic stride error will cancel each other out on a trial level when comparing both left and right circles. The horizontal shift of the lowest point of the back during trot does not appear to affect the VDS, which showed the lowest error among all keypoints.

The results support the previous validation of the groundline estimation and effect of handheld use of the same algorithm in a treadmill study^19^ and the previous keypoint precision and symmetry metrics agreement study under field conditions.^27^ The latter comparing RH algorithm detected keypoints with manually annotated keypoints showed lower stride‐ and trial‐level error on the circle than on the straight.^27^ The increased circle error in this study is attributed to the difference in anatomical location of the eye/forehead, the croup Mindiff error and the small circle size and suboptimal surface for lungeing as discussed. Another possible contributor is misalignment between the RH estimated groundline and the QS calibrated ground, which may be more pronounced on the circle than on the straight line.

Most validation studies of objective gait analysis systems have mainly reported trial‐level agreement with other reference methods, often limited to straight‐line recordings.^26^, ^38^, ^39^ Such results should be interpreted with caution, as averaging across many strides reduces random variability and improves the accuracy of the trial mean, but does not improve the underlying stride‐to‐stride precision.^40^ Additional stride‐level reporting, ideally without stride exclusion based on within‐trial variation, is ideal to provide a transparent and comparable assessment of both precision and accuracy. In the present study, agreement was evaluated at both stride and trial levels under straight‐line and circle conditions.

Our agreement at both stride and trial levels was comparable to that reported for a wireless inertial measurement system (EquiMoves®), validated against optical motion capture at the withers and pelvis (but not head) and widely used for research purposes.^38^, ^41^, ^42^, ^43^

The motion capture comparison of another 2D markerless computer vision algorithm (Sleip®) by Lawin et al.^26^ provides an important comparison. In that study, another smartphone‐based single‐camera method was compared with 3D multi‐camera optical motion capture on a straight runway using a tripod‐mounted phone, and they reported an overall trial mean deviation of 2.2 mm for head and 2.2 mm for pelvis after stride and trial exclusions but did not report stride‐level absolute errors. At the pelvis (croup), our trial‐level error of 1.1 mm was 50% lower and at the head (eye), our error of 1.4 mm was 36% lower, both differences being statistically significant at the 95% level, despite our use of a handheld phone and without stride exclusions based on algorithm variability. This indicates that handheld recordings are a viable alternative to tripod‐mounted setups used in other studies,^26^ supporting the practical application of RH and providing a foundation for future direct comparisons with systems such as Sleip®.

We also analysed lungeing on a circle, where we report trial‐level MAEs of about 2–3 mm for most keypoints and identified the croup Mindiff as an outlier, although the trial error of below 5 mm is still considered clinically useful as both circles will be analysed and compared in any clinical situation. To our knowledge, circle validation has not previously been reported for markerless computer vision systems.

The current algorithm relies on a strict side‐view approach, which may limit its applicability and user preference in some settings. However, this requirement also enables horse owners to record their horses independently during lungeing, without assistance from a second person.

Overall, the present work extends and complements previous studies of the same algorithm^19^, ^27^ and validates its performance on both straight‐line and circle gait analysis using a handheld setup.

## 5 CONCLUSION

This study validated a handheld smartphone‐based markerless computer vision system (RH) against a 3D multi camera optical motion capture reference (QS) for measuring vertical displacement symmetry at the eye, withers, back and croup during trot on a straight line and on a circle. The two streams were synchronised at frame level, processed with identical filtering, and compared by Maxdiff and Mindiff at stride level and at trial level. On the straight line the pooled stride errors were small, with MAEs of 3.6–3.9 mm when Maxdiff and Mindiff were combined, and keypoint specific errors of 5.1 mm for the eye, 4.3 mm for the withers and 3.0 mm for the croup. Aggregation to trial level further reduced errors to 1.4 mm for the eye, 1.4 mm for the withers and 1.1 mm for the croup. On the circle the pooled stride error was 5.5 mm and trial errors remained low for most keypoints. Across all analyses the mid back showed the lowest errors.

Future work could improve the croup stance phase detection on a circle. Comparison with other objective systems beyond motion capture will also be valuable.

Taken together, these results show that a handheld smartphone‐based approach can provide vertical displacement symmetry measures that agree closely with 3D multi‐camera motion capture under these study conditions. Precision was high on the straight line and remained well acceptable on the circle, with a single area for improvement at the croup Mindiff on the circle where anatomical definition differs between systems. No strides were excluded based on RH algorithm variation. The results support the use of the algorithm in both clinical gait analysis and research applications.

## FUNDING INFORMATION

This research was funded by Keydiagnostics ApS as part of the internal validation of their algorithm. No external grants or funding were received.

## CONFLICT OF INTEREST STATEMENT

Karsten Key, Jakob Kirkegaard and Katja Berg are affiliated with Keydiagnostics ApS, a company that provides a commercially available smartphone application ‘RealHorse’ for detecting asymmetry in horses. The computer vision algorithm developed and tested in this study is part of this product.

## AUTHOR CONTRIBUTIONS

**Karsten Key:** Conceptualization; methodology; data curation; writing – original draft; formal analysis; investigation; project administration. **Jakob Kirkegaard:** Writing – review and editing; software; data curation; methodology. **Katja Berg:** Investigation; data curation. **Kristian Ringkjær Andresen:** Investigation. **Sabrina Skov Hansen:** Writing – review and editing.

## DATA INTEGRITY STATEMENT

Karsten Key had full access to all the data in the study and takes responsibility for the integrity of the data and the accuracy of data analysis.

## ETHICAL ANIMAL RESEARCH

Local ethical approval was obtained in accordance with the university ethical guidelines (the Local Ethical and Administrative Committee of the University of Copenhagen, Department of Veterinary Clinical Sciences no. 2025‐012).

## INFORMED CONSENT

Written owner consent was obtained prior to inclusion in the study.

## Supporting information


**Figure S1.** Stride‐level Maxdiff and Mindiff aggregated data comparing RH and QS for the Withers on a straight line. The scatter plot in the top panel shows the paired values against the line of equality. The Bland Altman plot in the middle panel indicates a bias of −0.11 mm with 95% limits of agreement at −14.99 to +14.78 mm. The histogram of absolute differences shows a mean absolute error (MAE) of 4.31 mm. Observations include *n* = 5166 paired stride‐level metrics. Stride‐level LoA are indicative and should be interpreted descriptively because strides within a horse are not fully independent.

**Figure S2.** Stride‐level Maxdiff and Mindiff aggregated data comparing RH and QS for the Back on a straight line. The scatter plot in the top panel shows the paired values against the line of equality. The Bland Altman plot in the middle panel indicates a bias of 0.02 mm with 95% limits of agreement at −8.00 to +8.04 mm. The histogram of absolute differences shows a mean absolute error (MAE) of 2.78 mm. Observations include *n* = 5178 paired stride‐level metrics. Stride‐level LoA are indicative and should be interpreted descriptively because strides within a horse are not fully independent.

**Figure S3.** Stride‐level Maxdiff and Mindiff aggregated data comparing RH and QS for the Eye on a circle. The scatter plot in the top panel shows the paired values against the line of equality. The Bland Altman plot in the middle panel indicates a bias of −0.55 mm with 95% limits of agreement at −20.59 to +19.50 mm. The histogram of absolute differences shows a mean absolute error (MAE) of 6.40 mm. Observations include *n* = 4262 paired stride‐level metrics. Stride‐level LoA are indicative and should be interpreted descriptively because strides within a horse are not fully independent.

**Figure S4.** Stride‐level Maxdiff and Mindiff aggregated data comparing RH and QS for the Withers on a circle. The scatter plot in the top panel shows the paired values against the line of equality. The Bland Altman plot in the middle panel shows a bias of −0.19 mm with 95% limits of agreement at −17.73 to +17.35 mm. The histogram of absolute differences shows a mean absolute error (MAE) of 5.59 mm. Observations include *n* = 4674 paired stride‐level metrics. Stride‐level LoA are indicative and should be interpreted descriptively because strides within a horse are not fully independent.

**Figure S5.** Stride‐level Maxdiff and Mindiff aggregated data comparing RH and QS for the Back on a circle. The scatter plot in the top panel shows the paired values against the line of equality. The Bland Altman plot in the middle panel shows a bias of 0.25 mm with 95% limits of agreement at −15.42 to +15.93 mm. The histogram of absolute differences shows a mean absolute error (MAE) of 4.80 mm. Observations include *n* = 4700 paired stride‐level metrics. Stride‐level LoA are indicative and should be interpreted descriptively because strides within a horse are not fully independent.

**Figure S6.** Stride‐level Maxdiff and Mindiff aggregated data comparing RH and QS for the Croup on a circle. The scatter plot in the top panel shows the paired values against the line of equality. The Bland Altman plot in the middle panel indicates a bias of 0.59 mm with 95% limits of agreement at −18.96 to +20.15 mm. The histogram of absolute differences shows a mean absolute error (MAE) of 7.03 mm. Observations include *n* = 4692 paired stride‐level metrics. Stride‐level LoA are indicative and should be interpreted descriptively because strides within a horse are not fully independent.

**Figure S7.** Trial‐level Maxdiff and Mindiff aggregated error for the Withers on a straight line. The scatter plot shows paired trial means against the line of equality. The Bland Altman plot shows a bias of −0.18 mm with 95% limits of agreement −4.02 to 3.66 mm. The histogram of absolute differences shows a mean absolute error (MAE) of 1.41 mm. *n* = 116 paired values.

**Figure S8.** Trial‐level Maxdiff and Mindiff aggregated error for the Back on a straight line. The scatter plot shows paired trial means against the line of equality. The Bland Altman plot shows a bias of 0.02 mm with 95% limits of agreement −1.90 to 1.94 mm. The histogram of absolute differences shows a mean absolute error (MAE) of 0.77 mm. *n* = 116 paired values.

**Figure S9.** Trial‐level Maxdiff and Mindiff aggregated error for the Eye on a circle. The scatter plot shows paired trial means against the line of equality. The Bland Altman plot shows a bias of −0.89 mm with 95% limits of agreement −7.92 to 6.13 mm. The histogram of absolute differences shows a mean absolute error (MAE) of 2.75 mm. *n* = 48 paired values.

**Figure S10.** Trial‐level Maxdiff and Mindiff aggregated error for the Withers on a circle. The scatter plot shows paired trial means against the line of equality. The Bland Altman plot shows a bias of −0.50 mm with 95% limits of agreement −5.22 to 4.23 mm. The histogram of absolute differences shows a mean absolute error (MAE) of 1.77 mm. *n* = 48 paired values.

**Figure S11.** Trial‐level Maxdiff and Mindiff aggregated error for the Back on a circle. The scatter plot shows paired trial means against the line of equality. The Bland Altman plot shows a bias of 0.30 mm with 95% limits of agreement −3.26 to 3.85 mm. The histogram of absolute differences shows a mean absolute error (MAE) of 1.44 mm. *n* = 48 paired values.

**Figure S12.** Trial‐level Maxdiff and Mindiff aggregated error for the Croup on a circle. The scatter plot shows paired trial means against the line of equality. The Bland Altman plot shows a bias of 1.21 mm with 95% limits of agreement −8.44 to 10.86 mm. The histogram of absolute differences shows a mean absolute error (MAE) of 3.26 mm. *n* = 48 paired values.

## Data Availability

The data that support the findings of this study are openly available in Zenodo at https://zenodo.org/records/17177526.
