# Supplementary material for: Validation of a handheld smartphone markerless gait‐analysis tool using an estimated groundline in horses
Source: Equine Vet J. 2026 Jan 16;58(5):1493–504. doi: 10.1002/evj.70149 (PMC13447902; doi:10.1002/evj.70149)

Equine Veterinary Journal BEVA

Figure S11: Trial-level Maxdiff and Mindiff aggregated error for the Back on a circle. The scatter plot shows paired trial means against the line of equality. The Bland Altman plot shows a bias of 0.30 mm with 95% limits of agreement −3.26 to 3.85 mm. The histogram of absolute differences shows a mean absolute error (MAE) of 1.44 mm. n = 48 paired values.

Circle – Back – Combined MinDiff/MaxDiff error (n = 48)

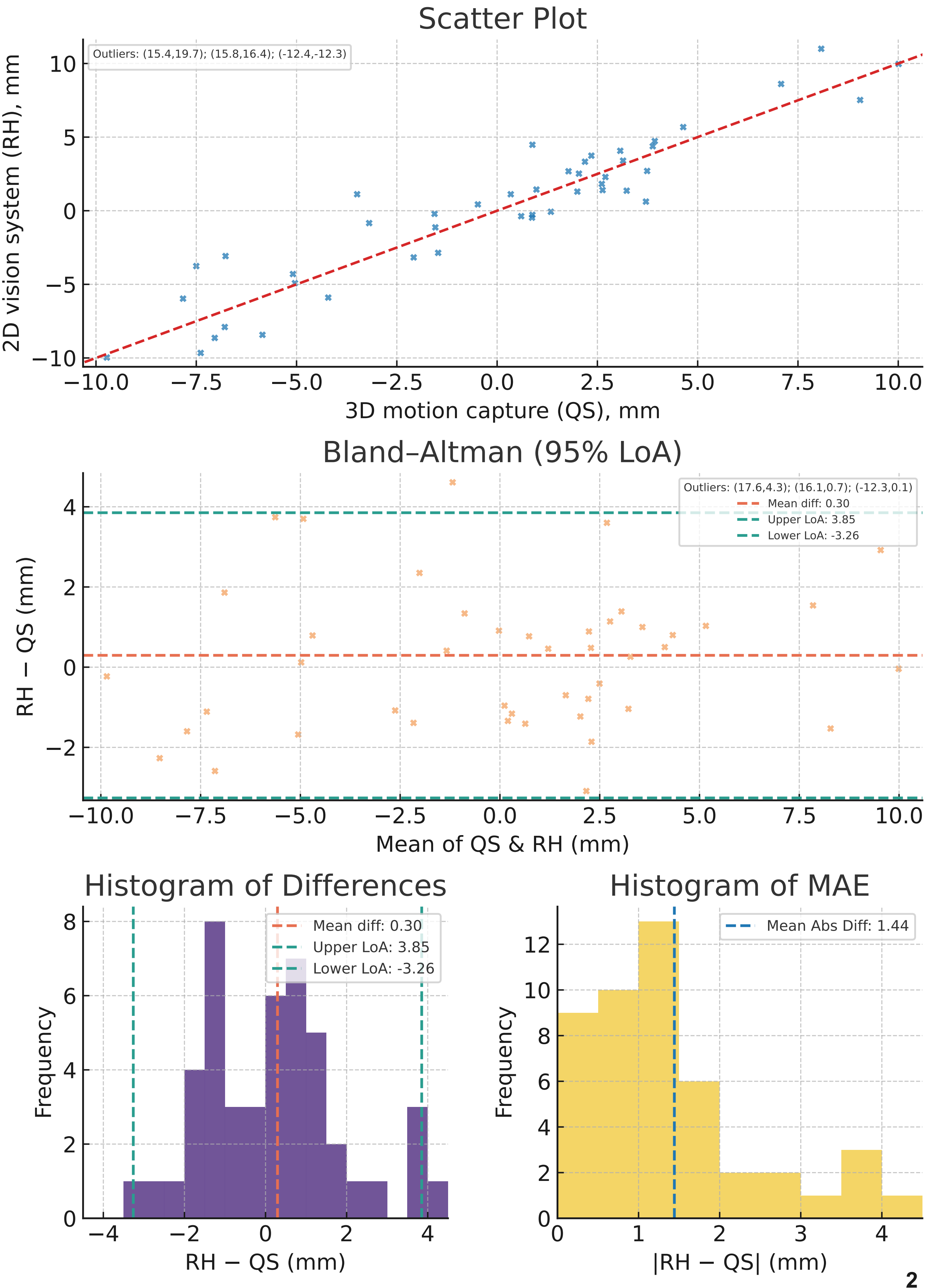

Supplement: Supplementary file 1 [file EVJ-58-1493-s001.pdf]
