# Supplementary material for: Validation of a handheld smartphone markerless gait‐analysis tool using an estimated groundline in horses
Source: Equine Vet J. 2026 Jan 16;58(5):1493–504. doi: 10.1002/evj.70149 (PMC13447902; doi:10.1002/evj.70149)

Equine Veterinary Journal BEVA

Figure S2: Stride-level Maxdiff and Mindiff aggregated data comparing RH and QS for the Back on a straight line. The scatter plot in the top panel shows the paired values against the line of equality. The Bland Altman plot in the middle panel indicates a bias of 0.02 mm with 95% limits of agreement at −8.00 mm to +8.04 mm. The histogram of absolute differences shows a mean absolute error (MAE) of 2.78 mm. Observations include n = 5178 paired stride-level metrics. Stride-level LoA are indicative and should be interpreted descriptively because strides within a horse are not fully independent.

Straight – Back – Combined MinDiff/MaxDiff errors (n = 5178)

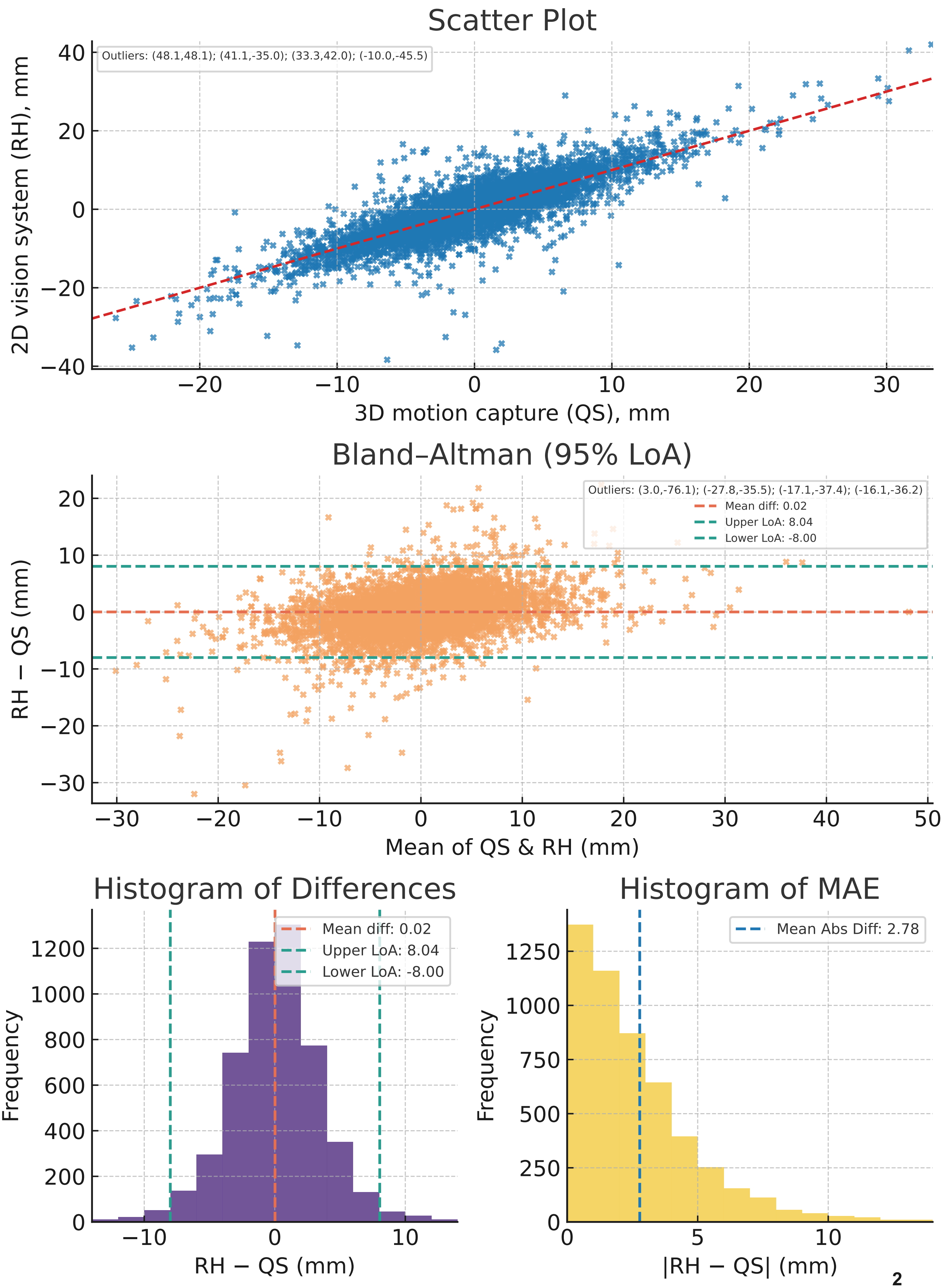

Supplement: Supplementary file 5 [file EVJ-58-1493-s005.pdf]
