# Supplementary material for: Validation of a handheld smartphone markerless gait‐analysis tool using an estimated groundline in horses
Source: Equine Vet J. 2026 Jan 16;58(5):1493–504. doi: 10.1002/evj.70149 (PMC13447902; doi:10.1002/evj.70149)
Supplement: Supplementary file 6 [file EVJ-58-1493-s006.pdf]

Equine Veterinary Journal BEVA

Figure S1: Stride-level Maxdiff and Mindiff aggregated data comparing RH and QS for the Withers on a straight line. The scatter plot in the top panel shows the paired values against the line of equality. The Bland Altman plot in the middle panel indicates a bias of −0.11 mm with 95% limits of agreement at −14.99 mm to +14.78 mm. The histogram of absolute differences shows a mean absolute error (MAE) of 4.31 mm. Observations include n = 5166 paired stride-level metrics. Stride-level LoA are indicative and should be interpreted descriptively because strides within a horse are not fully independent.

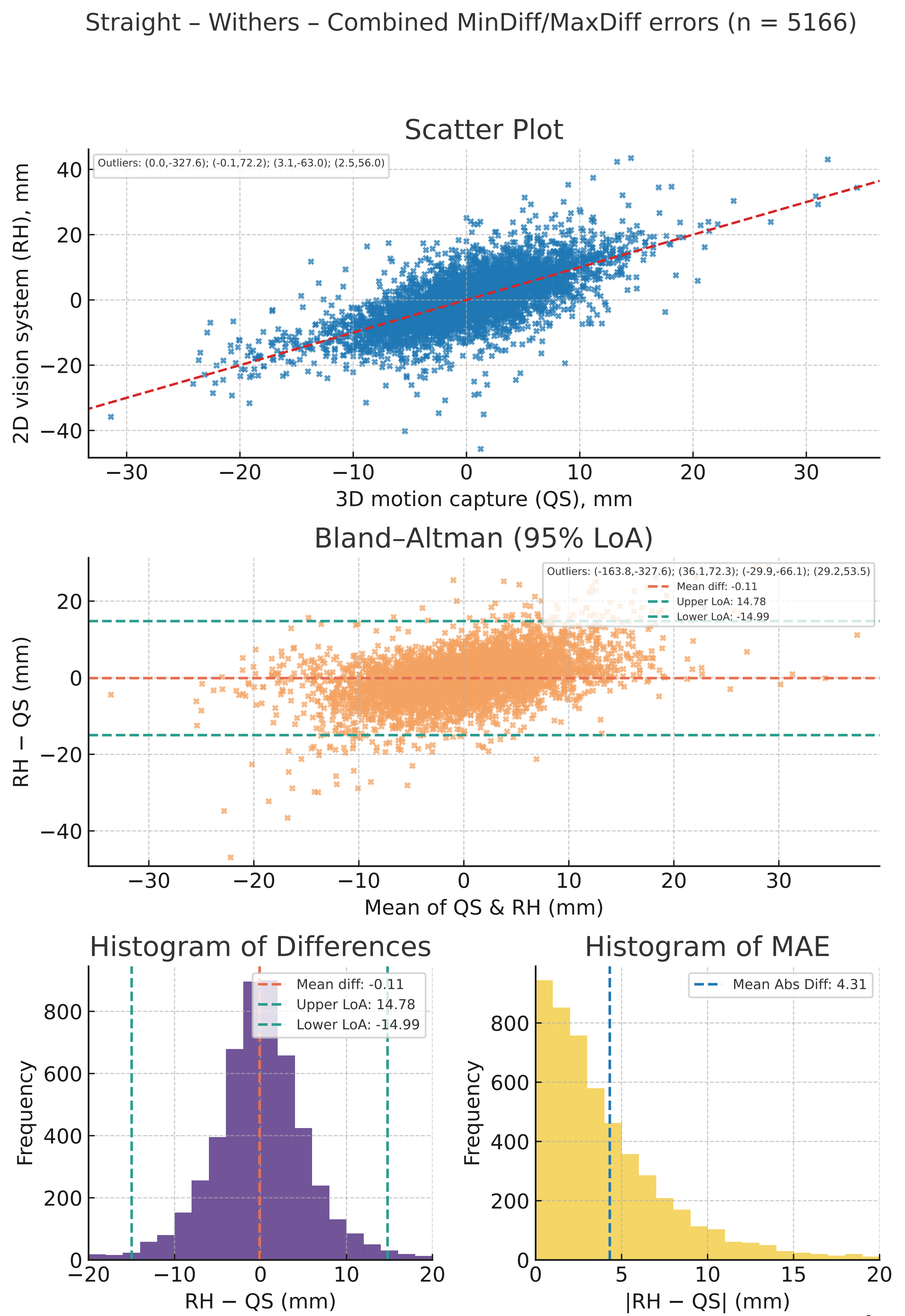
Straight – Withers – Combined MinDiff/MaxDiff errors (n = 5166)
Scatter Plot
Outliers: (0.0,-327.6); (-0.1,72.2); (3.1,-63.0); (2.5,56.0)
2D vision system (RH), mm
3D motion capture (QS), mm
Bland–Altman (95% LoA)
Outliers: (-163.8,-327.6); (36.1,72.3); (-29.9,-66.1); (29.2,53.5)
Mean diff: -0.11
Upper LoA: 14.78
Lower LoA: -14.99
RH − QS (mm)
Mean of QS & RH (mm)
Histogram of Differences
Mean diff: -0.11
Upper LoA: 14.78
Lower LoA: -14.99
Frequency
RH − QS (mm)
Histogram of MAE
Mean Abs Diff: 4.31
Frequency
|RH − QS| (mm)
