# Supplementary material for: Validation of a handheld smartphone markerless gait‐analysis tool using an estimated groundline in horses
Source: Equine Vet J. 2026 Jan 16;58(5):1493–504. doi: 10.1002/evj.70149 (PMC13447902; doi:10.1002/evj.70149)
Supplement: Supplementary file 7 [file EVJ-58-1493-s007.pdf]

Equine Veterinary Journal BEVA

Figure S9: Trial-level Maxdiff and Mindiff aggregated error for the Eye on a circle. The scatter plot shows paired trial means against the line of equality. The Bland Altman plot shows a bias of −0.89 mm with 95% limits of agreement −7.92 to 6.13 mm. The histogram of absolute differences shows a mean absolute error (MAE) of 2.75 mm. n = 48 paired values.

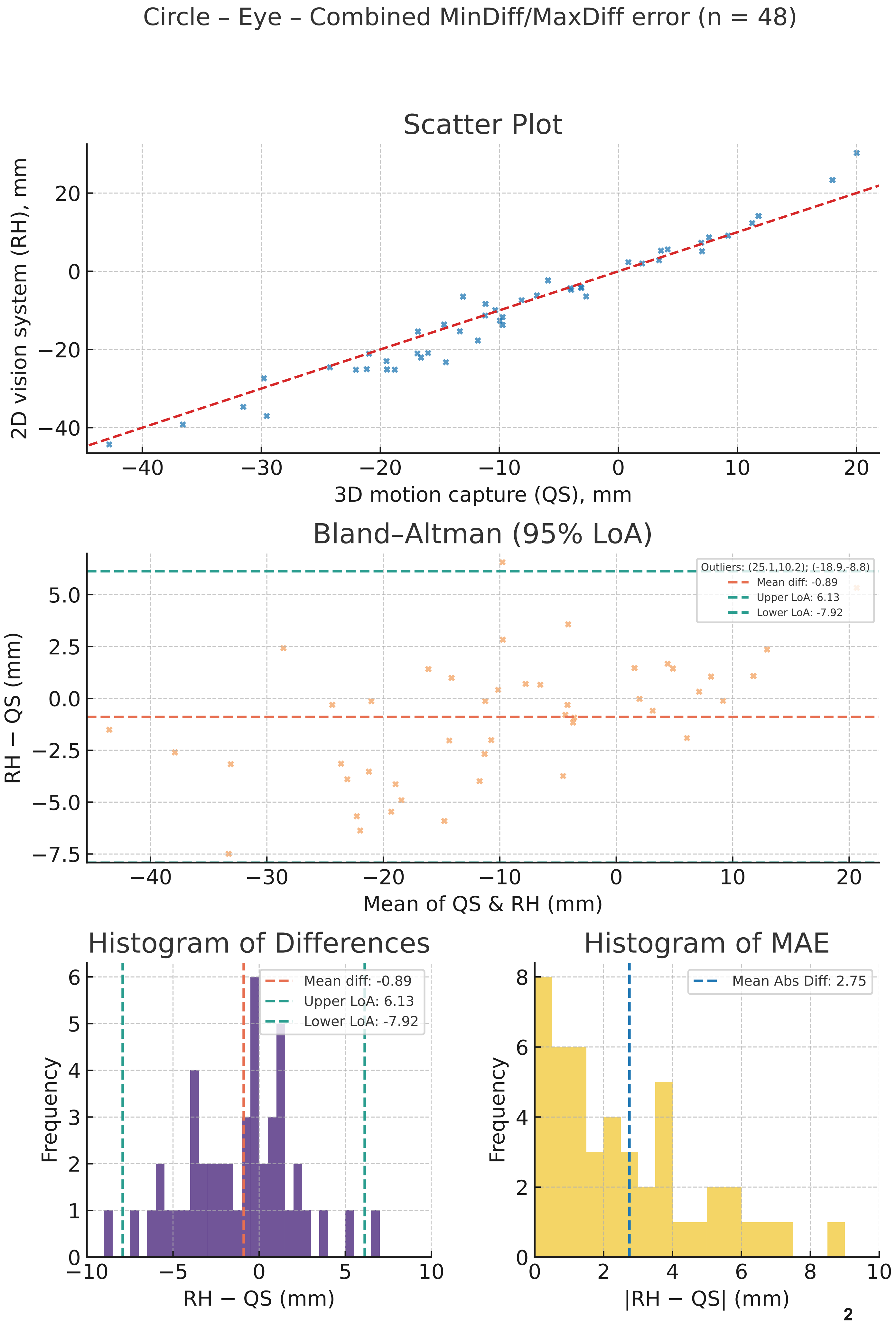
Circle – Eye – Combined MinDiff/MaxDiff error (n = 48)
Scatter Plot
2D vision system (RH), mm
3D motion capture (QS), mm
Bland–Altman (95% LoA)
Outliers: (25.1,10.2); (-18.9,-8.8)
Mean diff: -0.89
Upper LoA: 6.13
Lower LoA: -7.92
RH − QS (mm)
Mean of QS & RH (mm)
Histogram of Differences
Frequency
Mean diff: -0.89
Upper LoA: 6.13
Lower LoA: -7.92
RH − QS (mm)
Histogram of MAE
Frequency
Mean Abs Diff: 2.75
|RH − QS| (mm)
