# Supplementary material for: Validation of a handheld smartphone markerless gait‐analysis tool using an estimated groundline in horses
Source: Equine Vet J. 2026 Jan 16;58(5):1493–504. doi: 10.1002/evj.70149 (PMC13447902; doi:10.1002/evj.70149)
Supplement: Supplementary file 8 [file EVJ-58-1493-s008.pdf]

Equine Veterinary Journal BEVA

Figure S7: Trial-level Maxdiff and Mindiff aggregated error for the Withers on a straight line. The scatter plot shows paired trial means against the line of equality. The Bland Altman plot shows a bias of −0.18 mm with 95% limits of agreement −4.02 to 3.66 mm. The histogram of absolute differences shows a mean absolute error (MAE) of 1.41 mm. n = 116 paired values.

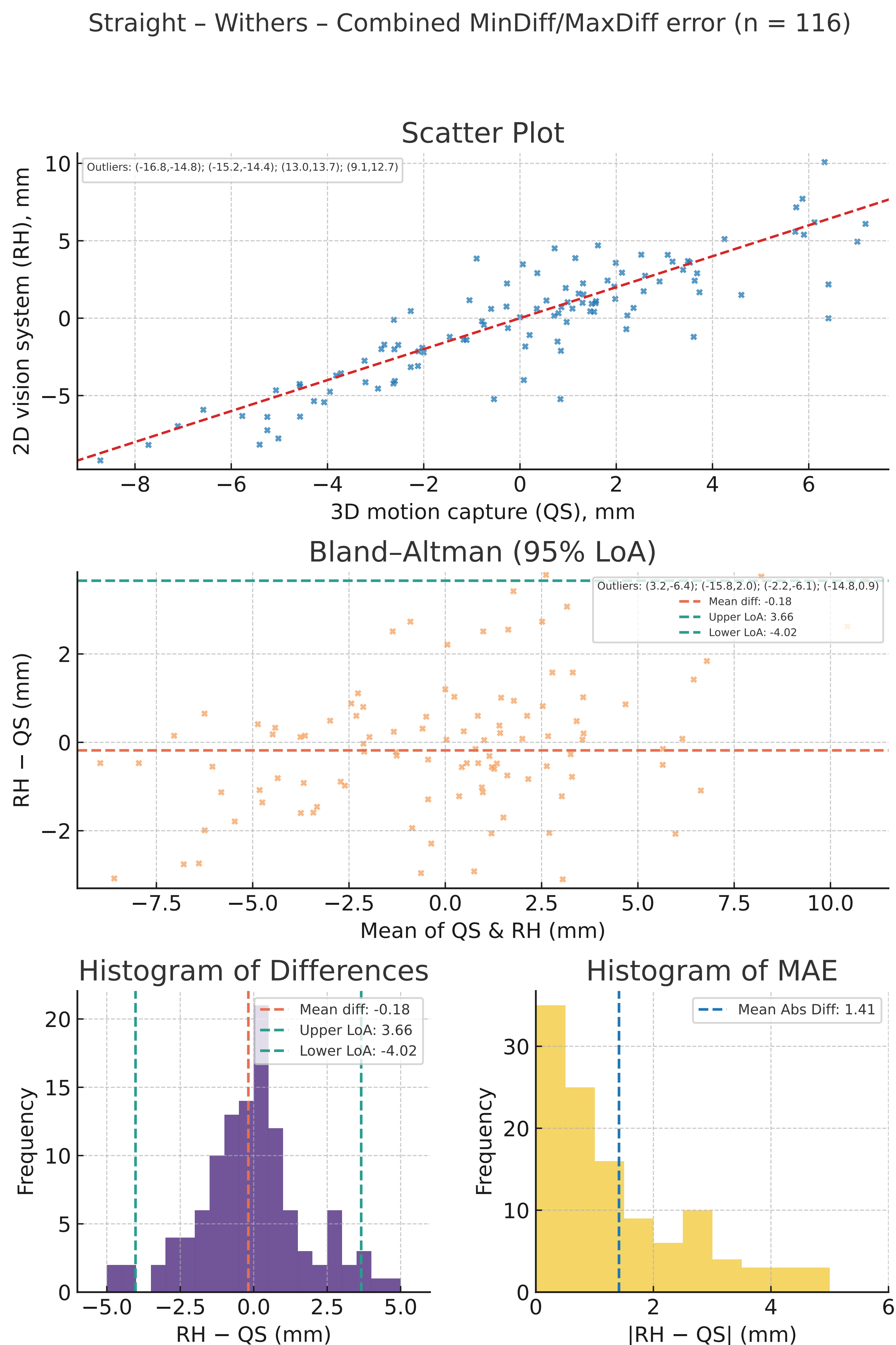
Straight – Withers – Combined MinDiff/MaxDiff error (n = 116)
Scatter Plot
Outliers: (-16.8,-14.8); (-15.2,-14.4); (13.0,13.7); (9.1,12.7)
2D vision system (RH), mm
3D motion capture (QS), mm
Bland–Altman (95% LoA)
Outliers: (3.2,-6.4); (-15.8,2.0); (-2.2,-6.1); (-14.8,0.9)
Mean diff: -0.18
Upper LoA: 3.66
Lower LoA: -4.02
RH − QS (mm)
Mean of QS & RH (mm)
Histogram of Differences
Mean diff: -0.18
Upper LoA: 3.66
Lower LoA: -4.02
Frequency
RH − QS (mm)
Histogram of MAE
Mean Abs Diff: 1.41
Frequency
|RH − QS| (mm)
