# Supplementary material for: Validation of a handheld smartphone markerless gait‐analysis tool using an estimated groundline in horses
Source: Equine Vet J. 2026 Jan 16;58(5):1493–504. doi: 10.1002/evj.70149 (PMC13447902; doi:10.1002/evj.70149)

Equine Veterinary Journal BEVA

Figure S10: Trial-level Maxdiff and Mindiff aggregated error for the Withers on a circle. The scatter plot shows paired trial means against the line of equality. The Bland Altman plot shows a bias of −0.50 mm with 95% limits of agreement −5.22 to 4.23 mm. The histogram of absolute differences shows a mean absolute error (MAE) of 1.77 mm. n = 48 paired values.

Circle – Withers – Combined MinDiff/MaxDiff error (n = 48)

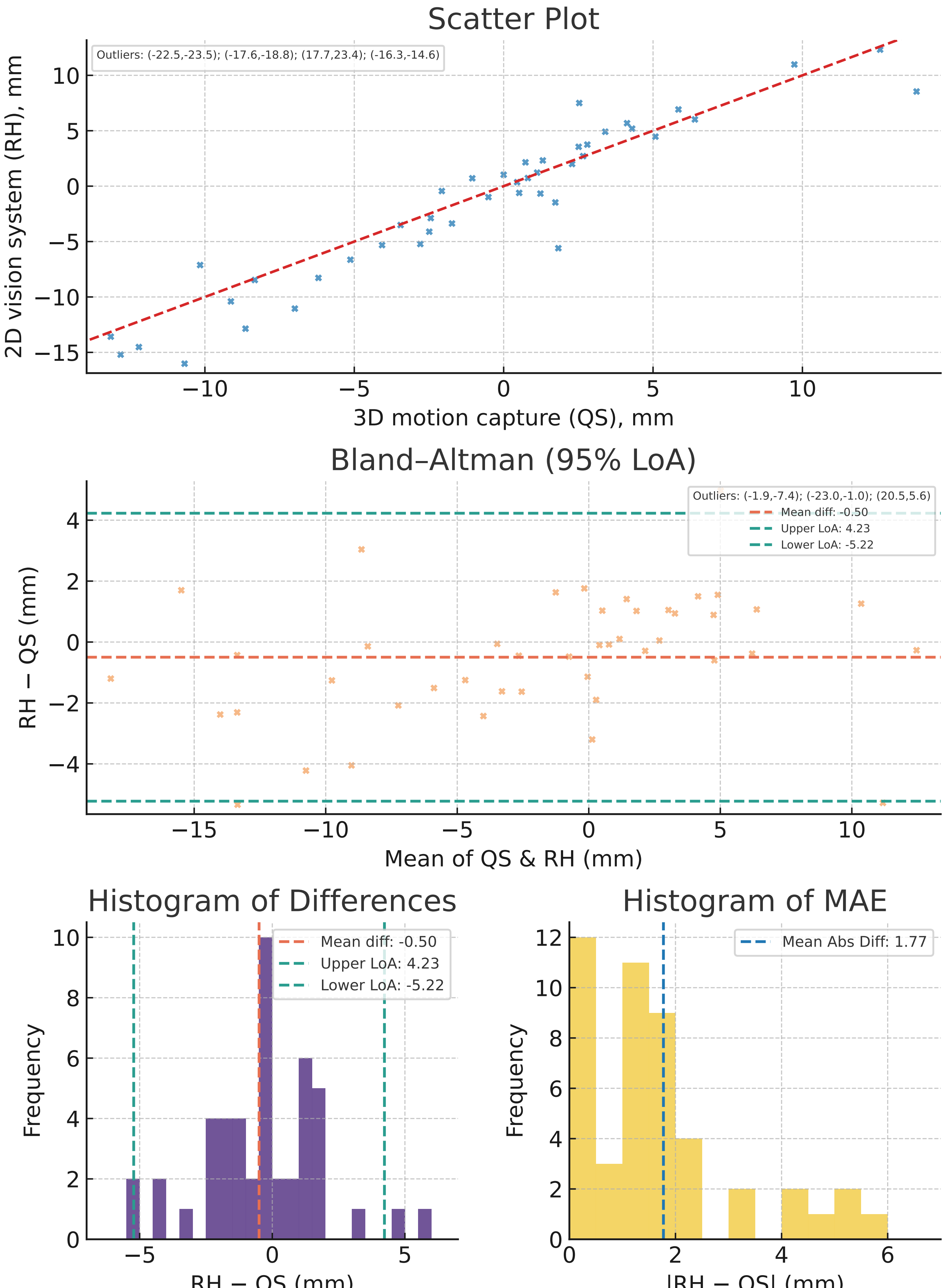

Supplement: Supplementary file 9 [file EVJ-58-1493-s009.pdf]
