# Supplementary material for: Validation of a handheld smartphone markerless gait‐analysis tool using an estimated groundline in horses
Source: Equine Vet J. 2026 Jan 16;58(5):1493–504. doi: 10.1002/evj.70149 (PMC13447902; doi:10.1002/evj.70149)

Equine Veterinary Journal BEVA

Figure S8: Trial-level Maxdiff and Mindiff aggregated error for the Back on a straight line. The scatter plot shows paired trial means against the line of equality. The Bland Altman plot shows a bias of 0.02 mm with 95% limits of agreement −1.90 to 1.94 mm. The histogram of absolute differences shows a mean absolute error (MAE) of 0.77 mm. n = 116 paired values.

Straight – Back – Combined MinDiff/MaxDiff error (n = 116)

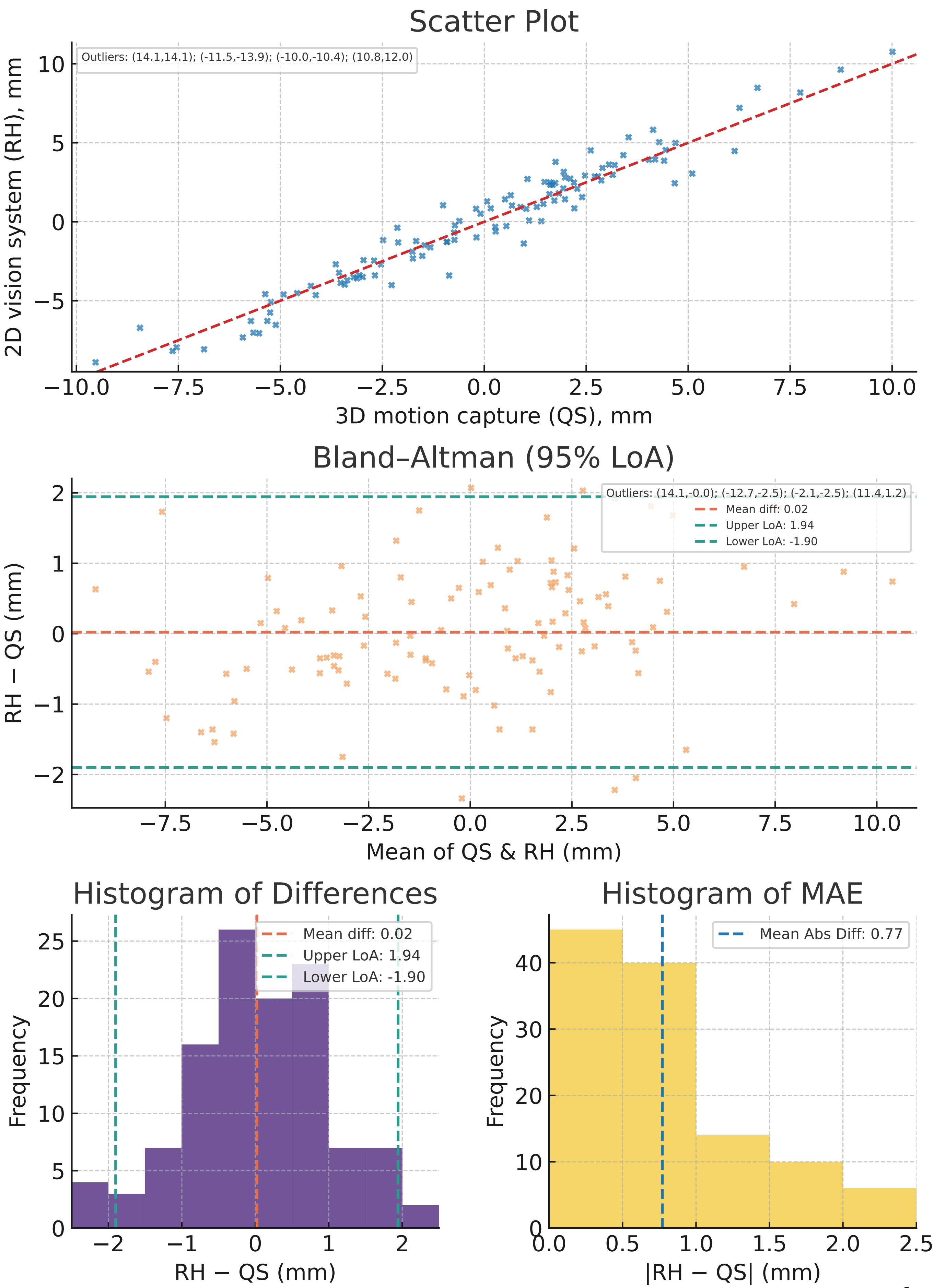

Supplement: Supplementary file 11 [file EVJ-58-1493-s011.pdf]
