# Supplementary material for: Validation of a handheld smartphone markerless gait‐analysis tool using an estimated groundline in horses
Source: Equine Vet J. 2026 Jan 16;58(5):1493–504. doi: 10.1002/evj.70149 (PMC13447902; doi:10.1002/evj.70149)
Supplement: Supplementary file 12 [file EVJ-58-1493-s012.pdf]

Equine Veterinary Journal BEVA

Figure S4: Stride-level Maxdiff and Mindiff aggregated data comparing RH and QS for the Withers on a circle. The scatter plot in the top panel shows the paired values against the line of equality. The Bland Altman plot in the middle panel shows a bias of −0.19 mm with 95% limits of agreement at −17.73 mm to +17.35 mm. The histogram of absolute differences shows a mean absolute error (MAE) of 5.59 mm. Observations include n = 4674 paired stride-level metrics. Stride-level LoA are indicative and should be interpreted descriptively because strides within a horse are not fully independent.

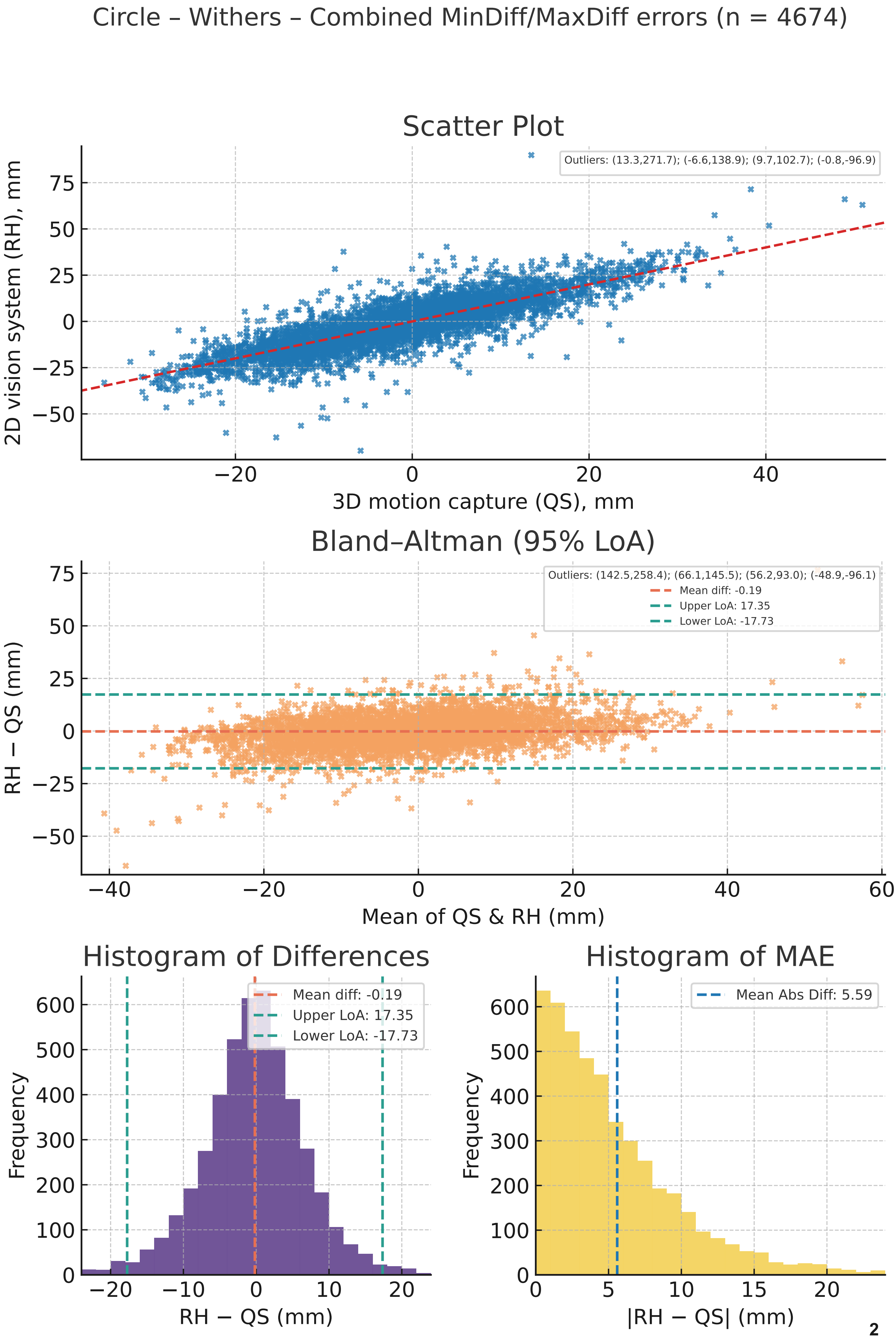
Circle – Withers – Combined MinDiff/MaxDiff errors (n = 4674)
Scatter Plot
Outliers: (13.3,271.7); (-6.6,138.9); (9.7,102.7); (-0.8,-96.9)
2D vision system (RH), mm
3D motion capture (QS), mm
Bland–Altman (95% LoA)
Outliers: (142.5,258.4); (66.1,145.5); (56.2,93.0); (-48.9,-96.1)
Mean diff: -0.19
Upper LoA: 17.35
Lower LoA: -17.73
RH − QS (mm)
Mean of QS & RH (mm)
Histogram of Differences
Mean diff: -0.19
Upper LoA: 17.35
Lower LoA: -17.73
Frequency
RH − QS (mm)
Histogram of MAE
Mean Abs Diff: 5.59
Frequency
|RH − QS| (mm)
